# Evaluating the Impact of a Novel Program to Address Acute Food Insecurity Among Cancer Patients

**DOI:** 10.3390/nu16244408

**Published:** 2024-12-23

**Authors:** Elliott M. Sina, Michael Rowe, Gina Mancuso, Gregory Garber, W. Kevin Kelly, Amy E. Leader

**Affiliations:** 1 Sidney Kimmel Medical College, Thomas Jefferson University, Philadelphia, PA 19107, USA; 2 Legacy of Hope, Philadelphia, PA 19130, USA; 3 Sidney Kimmel Comprehensive Cancer Center, Thomas Jefferson University, Philadelphia, PA 19107, USA

**Keywords:** food insecurity, cancer, quality of life, nutritional support, program evaluation

## Abstract

Introduction: Acute food insecurity (FI) significantly impacts cancer patients’ health, exacerbating physical and psychological burdens. While current interventions address chronic FI, acute cases remain undermanaged. Legacy of Hope, a Philadelphia-based non-profit, addresses this gap through its Emergency Patient Support Network (EPSN), offering free bi-weekly groceries to patients facing acute FI. Materials and Methods: The pilot study evaluated EPSN’s impact utilizing the Legacy of Hope Acute Food Insecurity (LOHAFI) survey, which was performed at baseline and two weeks post-intervention. The survey combines the Functional Assessment of Cancer Therapy—General (FACT-G7) and questions on food and financial security. Mean scores and frequencies were calculated. Results: Fifty patients (n = 50) completed the LOHAFI survey. The mean age was 55.1 years; 70% were female; 33 (66%) identified as Black. Two weeks after receiving groceries, patients reported a decrease in nausea (pre: 1.34; post: 1.18) and anxiety related to their cancer (pre: 2.49; post: 2.41) and an increase in the availability (pre: 1.70; post: 1.84) and consumption (pre: 2.26; post: 2.30) of healthy food. However, patient overall quality of life did not improve (pre: 13.14; post: 12.76). Conclusions: Legacy of Hope’s EPSN shows potential in alleviating acute FI among cancer patients, although larger studies are needed to fully assess its impact.

## 1. Introduction

A cancer diagnosis can be emotionally stressful and financially devastating for patients. A cancer diagnosis itself often induces psychological distress due to the uncertainty and fear associated with the disease prognosis and treatment outcomes [1]. Necessary cancer treatments can impose a substantial financial burden on patients as well, through direct medical expenses and indirect costs such as loss of income due to inability to work [2,3,4]. This combination of psychological stressors and financial strain may impact the overall well-being and quality of life of cancer patients [5].

Food insecurity (FI) is the condition of not having access to sufficient food, or food of adequate quality, to meet an individual’s basic health needs [6]. Food access, in this context, is defined as a sub-domain of food security specifically referring to the process of accessing food physically, socially, and economically. FI is intricately linked to social determinants of health (SDOH), which account for non-medical factors that influence health outcomes and quality of life. Notably, FI is a significant yet poorly understood social risk factor recognized among the cancer patient population [7].

It is thought that the prevalence of FI among cancer patients is substantially higher than in the general population, which tends to be roughly 10% of U.S. adults. A scoping review of 15 studies, although measuring food insecurity among cancer survivors rather than patients in active treatment, found that FI rates varied dramatically, from 4% to 84%; the median FI rate across the studies was 24% [8]. Often, a diagnosis of cancer can lead to FI, as seen in a state-wide survey that found that 10% of survivors who were food secure prior to their diagnosis became newly food insecure after their diagnosis [9]. These newly food insecure individuals were more likely to delay or go without necessary prescription medications and cancer treatments. Furthermore, because adherence to a cancer-focused nutritional regimen is critical to the success of treatment, FI in cancer patients is associated with adverse clinical outcomes, including further worsening of psychological distress and reduced treatment success [9,10,11].

Existing programs aimed at alleviating FI often target chronic rather than acute FI. Many food assistance programs, such as the Supplemental Nutrition Assistance Program (SNAP), are designed to provide ongoing support to individuals and families experiencing long-term food insecurity [12]. These initiatives are efficacious for reducing chronic FI but may not adequately address the immediate and urgent needs of families facing acute FI, particularly cancer patients who are suddenly unable to access nutritious foods due to financial hardships or treatment-related adverse effects [13]. Alternatively, hospital-based food pantries have been utilized among low-income cancer patients to meet immediate nutritional needs; however, such programs are institution-dependent, not widespread, and lack the comprehensive support required for long-term sustainability, often funded by philanthropy [14,15]. While various programs exist to mitigate FI, there remains a significant unmet need in addressing the issue of acute FI among cancer patients.

Legacy of Hope is a novel 501 (c)(3) non-profit organization based in Philadelphia, PA, that aims to address the issue of acute FI among cancer patients. Partnering with nearby cancer centers, local grocery stores, and the Philadelphia police department, Legacy of Hope’s Emergency Patient Support Network (EPSN) provides emergent and sustained nutritional support to cancer patients in acute financial distress. The EPSN’s efficacy and efficiency lie in its operational simplicity and integration into existing systems such as health networks and grocery chains. Upon receiving patient referrals from social workers and oncology dietitians at cancer centers to identify patients and families in need, Legacy of Hope enables cancer patients to receive one or more bi-weekly grocery deliveries until their acute FI needs are addressed. The EPSN began in February 2020; in 2023, Legacy of Hope’s EPSN served 507 cancer patients, supported 1324 patients’ household family members, and provided 85,176 meals.

The purpose of this study was to develop and implement the first evaluation of Legacy of Hope’s EPSN on cancer-related symptoms such as anxiety, pain, and nausea, as well as on food and nutritional aspects such as the quantity of food in the home and worry about not having nutritional food for one’s family. We aimed to elucidate the impact of the Legacy of Hope program on cancer patients in Philadelphia facing FI from both a programmatic and efficacy perspective.

## 2. Materials and Methods

### 2.1. FI Referral Process

The EPSN is a quickly scalable platform for health systems to address acute FI in oncology populations. The referral process begins with oncology social workers, who are often most aware of patient needs beyond their medical care. Social workers at every major cancer center in Philadelphia are given acute FI criteria to qualify for the EPSN: the patient must be a resident of Philadelphia, have experienced hunger or have been without a sufficient quantity of affordable and nutritious food in the last month, and/or it is reasonable to expect the patient or their family will experience hunger or go without a sufficient quantity of such food within the next month, and that the patient’s financial distress is the function of a cancer diagnosis. Subsequently, grocery orders created by oncology dietitians are placed with local grocery stores and delivered by Philadelphia police to the homes of patients within hours of the referral in the most urgent cases. Working with existing grocery chains leverages the network and experience of grocery executives and eliminates many costs, such as the need for warehouses and staff to manage large amounts of food. A description of the food included in a standard delivery order is provided in the Supplementary Materials.

### 2.2. Tudy Eligibility Criteria and Sample Size

Patients at either Thomas Jefferson University’s Sidney Kimmel Cancer Center, the University of Pennsylvania’s Abramson Cancer Center, and Temple University’s Fox Chase Cancer Center were eligible for inclusion in this study if they received groceries from Legacy of Hope’s EPSN. Patients were all over the age of 18, as the three cancer centers only treat adult cancer patients. Patients were able to complete the survey in English or Spanish. The goal of this research was to preliminarily evaluate the impact of the EPSN prior to any larger-scale study in the future. While not powered to detect significant differences between pre- and post-test scores, we believe that the sample of 50 patients provides insight into the impact of the EPSN and valuable data on effect sizes for future, larger studies.

### 2.3. Survey Development

The Legacy of Hope Acute Food Insecurity (LOHAFI) survey is a combination of questions incorporating all seven quality of life factors included in the validated Functional Assessment of Cancer Therapy (FACT-G7) [16] in addition to four questions developed by the study team that may more directly target topics related to food and financial insecurity. These four questions covered topics related to the cost of food (“I worry whether I can afford food for me and my family” and “I have had to make trade-offs to buy food for me and my family”), quantity of food (“I have enough food for me and my family to eat”), and nutritious food consumption (“My family and I eat healthy food”). Demographic questions included the patient’s age, race/ethnicity, gender, cancer diagnosis, size of the household, home address, and the number of children living in the house. The Wisconsin Area Deprivation Index (ADI), which has been validated by previous epidemiologic studies [17], was used to evaluate the socioeconomic status of the neighborhoods where each participant resided.

### 2.4. Data Collection

The LOHAFI survey was developed via the RedCap platform (Version 14.6.8) and administered to patients via text message at the time of the first grocery delivery and subsequently two weeks following the delivery. A two-week time interval was selected to assess changes in patient outcomes in order to specifically target the question of acute rather than chronic FI. When a patient was scheduled to receive a grocery delivery, a representative of the EPSN contacted the patient via phone call to confirm the time of delivery. During this phone call, the representative informed the patient of this study, obtained verbal consent, and sent the patient the Time 1 LOHAFI survey via text message. At the two-week follow-up time interval, a text message is automatically sent to the patient to complete the Time 2 LOHAFI survey. A representative of Legacy of Hope also called the patient to remind them to complete the follow-up survey after it was automatically sent. No financial incentives were provided for patients to complete the surveys. The survey was available in English or Spanish.

Data was collected via RedCap. Only patients with complete data at both time points were included in the final dataset. Mean scores and frequencies were calculated to evaluate the changes in the outcome measures of interest collected via the LOHAFI survey. A summary score for the 7 FACT-G was calculated at each time point. Paired samples t-tests were used to evaluate the changes in survey item responses between the two time points. Data analysis was conducted via SPSS version 29.0.2.0. This study received approval from the Institutional Review Board at Thomas Jefferson University.

## 3. Results

### 3.1. Participant Characteristics

We enrolled patients in this study until we accrued 50 patients with complete data (Table 1). This required us to reach out to 196 patients who were served by Legacy of Hope’s EPSN between June 2023 and May 2024. Among the 50 cancer patients included in this study, 35 (70%) were female, 33 (66%) identified as Black, and 47 participants (94%) identified as non-Hispanic. Participants ranged in age from 28 to 85 years, with a mean age of 55.1 years. Breast cancer was the most common diagnosis (38%), followed by blood cancers (16%). Slightly more than half of patients (54%) were receiving active curative treatment, while 42.6% were in palliative care and 3.4% were in hospice. Most participants (74%) reported having no children, and household size varied from 1 to 10 family members, with a mean of 2.47 family members. Twenty-nine participants (58%) had never received groceries from EPSN before, while 20 participants (40%) had previously received grocery deliveries. Roughly one-quarter (28%) of participants resided in a neighborhood of high deprivation, while the majority (42%) resided in a neighborhood of moderate to average deprivation.

### 3.2. Survey Results

We assessed various quality of life and food insecurity measures at the time of grocery delivery and 2 weeks thereafter (Table 2). Patients reported improvements in nausea two weeks following grocery delivery (pre-delivery: 1.34; post-delivery: 1.18). Patients also reported a decrease in the level of anxiety regarding their prognosis (pre-delivery: 2.49; post-delivery: 2.41). Patients reported increased agreement with the sentiment that they have enough food for themselves and their family to eat (pre-delivery: 1.70; post-delivery: 1.84) and that they eat healthy food (pre-delivery: 2.26; post-delivery: 2.30). However, a summary score for FACT-G7-related outcome measures regarding sleep quality, satisfaction with life, energy levels, and pain levels did not demonstrate an improvement following grocery deliveries (pre-delivery: 13.10; post-delivery: 12.66). Additional outcome measures evaluating patients’ level of worry regarding the ability to afford foods, as well as the necessity to make trade-offs to afford foods, also did not demonstrate an improvement when compared to baseline.

## 4. Discussion

This study is one of the first in the literature to assess outcome measures related to the impact of an intervention on cancer patients’ experiences with acute FI. Our findings suggest the potential impact of the Legacy of Hope EPSN in addressing FI among cancer patients. Most notably, we observed increases in patients reporting that they had enough food for their family and that they had access to healthy food. Patients also reported decreases in anxiety and nausea, two common elements of cancer care that greatly impact quality of life. Previous studies have evaluated the impact of FI interventions in cancer patient populations, yet none aimed to understand the nuanced and multi-faceted impact on access to healthy foods and the impact on well-being [18,19]. A recently published randomized controlled trial that tested interventions for food-insecure cancer patients found significant changes in measures that were similar to those investigated in our study, such as anxiety and food availability [20]. The LOHAFI survey combined elements of validated tools like the FACT-G7 with specific questions targeting food and financial security, making it uniquely suited for evaluating the impact of acute FI interventions for cancer patients. Unlike alternative tools, the LOHAFI survey aimed to capture the immediate psychological distress and quality of life changes that the Legacy of Hope program aims to address.

The unique benefit and novelty of Legacy of Hope compared to alternative food security service models is its ability to meet the urgent nutritional needs of cancer patients. Existing programs targeting FI are designed to remedy chronic and persistent FI but are not designed to address the highly fluid nature of acute FI. For example, the recent FINDING-Food intervention study demonstrated the efficacy of using informational memos to reduce FI among individuals using food pantries, yet such programs focus on longer-term food security solutions and do not address the issue of acute FI [21]. It is important to note the EPSN is not intended to supplant programs that address chronic food insecurity, such as SNAP; rather, this network was designed specifically to address the complex nature of acute food insecurity.

The findings in the present study are preliminary. The lack of improvement in all of the measures may reflect various factors in the patients’ cancer journey unrelated to the grocery deliveries provided by Legacy of Hope. Such factors may include concurrent medical treatments that can significantly affect patients’ physical and psychosocial wellbeing, potentially obscuring the effects of food security interventions. Additionally, the degree to which patients experienced adverse SDOHs prior to their cancer diagnosis may also impact the survey’s capacity to measure acute changes related to Legacy of Hope’s grocery deliveries. Changes in psychosocial support from both family members and other social services, such as counseling and financial aid programs, may also play a critical role in mitigating cancer-related anxiety, thus confounding outcomes potentially related to the grocery deliveries. Furthermore, it is possible that the natural progression in a patient’s adaptation to their condition or natural daily variability in a patient’s mood can influence their perceived quality of life at the time of survey completion. These reasons, and others associated with the daily life changes of a cancer patient, underscore the complexity of isolating the effects of an acute grocery delivery program alone.

Future evaluations of Legacy of Hope’s impact on cancer patients should consider additional measures that may be relevant. For example, tracking nutritional and inflammatory biomarkers associated with cancer could provide a more direct assessment of a patient’s physiologic response to consuming healthier foods [22,23,24]. Furthermore, integrating anthropometric measures such as BMI or body fat percentage could be used to monitor changes in patients’ physical health status, serving as a quantitative measurement of potential cachexia or obesity [25]. Dietary intake assessments, such as 24-h dietary recall tools, could provide more detailed insights into the quality and quantity of consumption of the foods provided by Legacy of Hope, strengthening the association between the intervention and patients’ actual dietary behaviors. Beyond physiologic measures, collecting and analyzing economic data such as changes in patients’ healthcare expenditures and financial statements throughout the course of their treatment and before and after Legacy of Hope’s intervention could provide insights into the economic impact and cost-effectiveness of the program. Higher-level clinical outcome measures, such as re-hospitalization rates and treatment adherence, could be collected to further establish a connection to a patient’s nutritional status.

Legacy of Hope’s model possesses significant potential for scalability and applicability to other cancer centers whose patients also face acute FI. The flexibility and rapid responsiveness of EPSN, which collaborates with local businesses and social workers at affiliated cancer centers, allows for rapid adaptation to the particular nutritional needs and care protocols associated with a range of specific oncologic conditions. Theoretically, extending Legacy of Hope’s model could benefit patients with any condition that becomes financially and/or physically debilitating. The unique and novel value of Legacy of Hope’s model is twofold: firstly, its ability to assist patients experiencing acute FI emergencies within 24 h, and second, its ability to tailor grocery deliveries to match the specific dietary requirements for conditions in which specific dietary management plays a critical role in therapeutic success and patient outcomes.

Moreover, the infrastructure already developed for cancer patients provides a robust framework that can be adjusted to specific requirements of other patient populations in an effort to enhance overall access to necessary nutritional support for patients. The scalability of Legacy of Hope’s EPSN lies in leveraging existing resources such as social workers, grocery stores, and delivery services, without the need for additional hires. By efficiently integrating hospital referrals, grocery orders, and delivery connections, the EPSN is able to unite disjointed systems. Partnerships with local entities enable a relatively small number of volunteers or staff members to manage thousands of deliveries, demonstrating the capacity of ESPN to be effectively replicated in other cities.

The present pilot study is not without limitations. Notably, the small sample size of 50 patients was the target goal for this pilot study; the small sample size may limit the generalizability of our findings, as this study was not designed to detect statistical significance. Additionally, cancer patients were recruited from Philadelphia, PA, also potentially limiting the generalizability of the findings to patients across the country. While the FACT-G7 tool provided a useful starting point for evaluating health-related quality of life in the cancer patient population, it is conceivable that the grocery deliveries did not directly address many of the tool’s targeted questions, suggesting a potential misalignment with measuring the intervention’s impact. Additional aforementioned confounders, such as changes in medical treatments, social support networks, and financial state that may have occurred during a patient’s cancer journey, may have influenced our LOHAFI survey outcomes. Community-level factors such as patients’ access to other long-term food assistance programs were also not accounted for in the present study. Additionally, the research team’s decision to instate a two-week follow-up period in order to specifically capture short-term changes in the outcome measures may not have captured the immediate beneficial effects of Legacy of Hope’s grocery deliveries as completely as a 7- or 10-day interval, as there may have been a delay between the time of grocery utilization and completion of the second survey. Notably for future studies, the lack of a control group in our study limits the ability to evaluate changes attributable to the intervention versus external confounding factors. In the future, larger studies should incorporate a control group to facilitate both between-group and within-group comparative analyses. However, our preliminary findings demonstrate that a model combining automated survey notifications and personalized phone calls enhances patient responsiveness. Future investigations should further investigate enhanced patient outreach methods to increase the rate of patient response.

## 5. Conclusions

In summary, Legacy of Hope is a novel program that addresses the significant and urgent need for food security among cancer patients through immediate and targeted grocery deliveries. Preliminary data from the present pilot study suggest that Legacy of Hope addresses the issue of acute FI and has a quantifiable impact on patients’ physical and emotional conditions. The high volume of grocery deliveries demonstrates the extensive reach of the Legacy of Hope program. With further investigation, Legacy of Hope’s EPSN may prove to be a sustainable, efficacious, and replicable model that can be implemented in other cities through partnerships with local hospital systems.

## Figures and Tables

**Table 1:** Patient characteristics (n = 50).

Characteristic	N	%
**Sex**		
Male	15	30
Female	35	70
**Age, Mean (SD) in Years**	55.1 (12.4)	
**Race**		
Asian	2	4
Black	33	66
White	10	20
Other	5	10
**Ethnicity**		
Non-Hispanic	47	94
Hispanic	3	6
**Cancer Type at Diagnosis**		
Blood Cancer (leukemia, lymphoma, myeloma, etc.)	8	16
Breast	19	38
Colon/Rectal/GI	4	8
Lung	4	8
Prostate/GU	2	4
Stomach	2	4
Other	11	22
**Household Size, Mean (SD)**	2.5 (1.6)	
**Children Living in Household**		
None	37	74
One Child	9	18
2–3 Children	4	4
**Previous Legacy of Hope Grocery Delivery**		
No	29	60
Yes	20	40
**Area Deprivation Index**		
Deciles 1–5 (least disadvantaged)	14	30
Deciles 6–9	20	42
Decile 10 (most disadvantaged)	13	28

Caption: Demographic and clinical characteristics of study participants, including sex, cancer types, household size, and socioeconomic context (N = 50). N = number of participants. SD = standard deviation.

**Table 2:** Legacy of Hope Acute Food Insecurity (LOHAFI) survey outcomes (n = 50).

	Survey Question	Time 1 (Day 0)		Time 2 (Day 14)	
		Mean	SD	Mean	SD
FACT-G7					
Q1	I worry that my condition will become worse.	2.49	1.22	2.41	1.33
Q2	I am sleeping well.	1.94	1.06	1.92	1.09
Q3	I am able to enjoy life.	2.08	1.10	1.98	1.15
Q4	I am content with the quality of my life right now.	1.60	1.44	1.38	1.32
Q5	I have a lack of energy.	2.32	1.17	2.54	1.09
Q6	I have pain.	2.34	1.33	2.43	1.34
Q7	I have nausea.	1.34	1.10	1.18	1.24
SUM	FACT-G7 Score *	13.10	5.18	12.66	5.05
Food and Nutritional Questions					
Q8	I worry about whether I can afford food for me and my family.	2.56	1.23	2.62	1.16
Q9	I have enough food for me and my family to eat.	1.70	1.00	1.84	0.98
Q10	I have had to make trade-offs to buy food for me and my family.	0.96	1.16	0.98	1.17
Q11	My family and I eat healthy food.	2.26	1.07	2.30	0.86

Caption: Legacy of Hope Acute Food Insecurity survey outcomes at baseline (Timepoint 1, Day 0) and follow-up (Timepoint 2, Day 14) for study participants (N = 50). (*) The FACT-G7 Score includes reverse scoring of FACT-G items # 1, 5, 6, and 7. N = number of participants. SD = standard deviation.

## Data Availability

The data that support the findings of this study are available upon request from the corresponding author, E.M.S. The data are not publicly available due to their containing information that could compromise the privacy of research participants.

## Supplementary Materials

The following supporting information can be downloaded at: https://www.mdpi.com/article/10.3390/nu16244408/s1. Table S1: Items included in one standard delivery order.
