# Supplementary material for: Evaluating the Impact of a Novel Program to Address Acute Food Insecurity Among Cancer Patients
Source: Nutrients. 2024 Dec 23;16(24):4408. doi: 10.3390/nu16244408 (PMC11679311; doi:10.3390/nu16244408)
Supplement: Supplementary file 1 [file nutrients-16-04408-s001.zip › nutrients-3348689-supplementary.pdf]

Supplementary Table S1

Standard Grocery Delivery Order
● 52 fl oz Tropicana Original No Pulp Orange Juice<br>● 16 oz Ortega Whole Wheat Tortillas<br>● 12 oz Thomas' 100% Whole Wheat English Muffin, 6 count<br>● 11.5 oz Quaker Oatmeal - Variety - Lower Sugar - 10 Packets<br>● 1 gallon Milk Fat Free<br>● 18 ct White Eggs, Large, 18 ct<br>● 18 ct Thin Sliced Sharp Cheddar Cheese<br>● 5 oz Jones Dairy Farm Golden Brown Turkey Sausage Links<br>● 1.3 lb Perdue Fresh Cuts Thin Sliced Chicken Breast<br>● 1 oz Perdue Ground Turkey - Lean<br>● 2 lb Carolina Rice - Enriched Extra Long Grain<br>● 1 Can of Tuna<br>● 15.25 oz Del Monte Corn - Whole Kernel Golden Sweet No Salt Added<br>● 19 oz Progresso Gluten Free Lentil with Roasted Vegetables Soup<br>● 19 oz Progresso Vegetable Classics Tomato Basil Soup<br>● 16 oz Ronzoni Spaghetti<br>● 14.5 oz Diced Tomatoes In Tomato Juice<br>● 32 oz Swanson® Vegetable Broth<br>● Uncle Ben's Ready Rice: Spanish Style, 8.8oz<br>● 15.5 oz Wholesome Pantry Organic Black Beans<br>● 3 ct Andy Boy Romaine Hearts<br>● 16 oz Bowl & Basket Baby Spinach<br>● 1 bunch Celery Bunch<br>● 18 oz Fresh Blueberries<br>● Fresh Cucumber<br>● 1 lb Fresh Strawberries, 1lb<br>● 9 oz Green Zucchini, 1 ct x2<br>● Hass Avocado x2<br>● 5 oz Honeycrisp Apple, 1 ct x6<br>● Lime1ctx3<br>● Navel Orange x5<br>● 6 oz Red Bell Peppers, 1 ct x4<br>● 1 lb Carrots<br>● 24 oz Baby Bella Mushrooms<br>● 0.5 lb Sweet Potato, 1 ct x4<br>● 8ozTomatoOnTheVine,1ctx3<br>● 4 oz Yellow Banana, 1 ct x6<br>● 5.5 oz Food Should Taste Good Multigrain Tortilla Chips

Caption: Items included in one standard delivery order.
